# Development of the PARMS Marker of the Dominant Genic Male Sterility (DGMS) Line and Its Utilization in Rapeseed (*Brassica napus* L.) Breeding

**DOI:** 10.3390/plants11030421

**Published:** 2022-02-03

**Authors:** Zhen Li, Rong Yuan, Miao Wang, Meiyan Hong, Li Zhu, Xiaofei Li, Ruixing Guo, Gang Wu, Xinhua Zeng

**Affiliations:** 1 School of Agriculture, Jinhua Polytechnic, Jinhua 321007, China; tulip0909@126.com; 2 Key Laboratory of Biology and Genetic Improvement of Oil Crops, Oil Crops Research Institute of the Chinese Academy of Agricultural Sciences, Ministry of Agriculture, Wuhan 430062, China; yuanrong@caas.cn (R.Y.); 13627171803@163.com (M.W.); mojiezuohmy@hotmail.com (M.H.); zhuli02@caas.cn (L.Z.); lixiaofei@caas.cn (X.L.); grx808@163.com (R.G.)

**Keywords:** *Brassica napus*, dominant genic male sterility, PARMS marker, MAS

## Abstract

The 8029AB line is a dominant genic male sterility (DGMS) two-type line in *Brassica napus* L., which can be used in a three-line approach for the seed production of rapeseed hybrids. Genetic analyses have demonstrated that the sterility of 8029A is controlled by a single dominant nuclear gene (*BnMS5^e^*) interacting with one recessive gene (*BnMS5^c^*). Six pairs of penta-primer amplification refractory mutation system (PARMS) markers were designed according to the sequence of *BnMS5^a^*, *BnMS5^c^* and *BnMS5^e^*. Two pairs of these PARMS markers were successfully identified and validated. The PARMS markers MS5-1Fc/MS5-1Ft/MS5-1R12 could distinguish *BnMS5^c^* from *BnMS5^a^*/*BnMS5^e^*, and the PARMS markers MS5-2Ft/MS5-2Fa/MS5-1R12 could genotype *BnMS5^a^* and *BnMS5^c^*/*BnMS5^e^*. The combination of these two pairs of PARMS markers could be used to identify the presence or absence of *BnMS5^a^/BnMS5^c^/BnMS5^e^* effectively. Consequently, marker-assisted selection can be carried out in the early generation to shorten the breeding period and improve the breeding efficiency.

## 1. Introduction

Rapeseed is the second most important oilseed crop in the world [1]. Heterosis utilization has become an important strategy to increase the productivity of rapeseed [2,3]. Remarkable heterosis had been well demonstrated in rapeseed [4]. Male sterility has been proved to be an economical and effective way to utilize heterosis in crops. At present, the main types of male sterility used worldwide in *Brassica napus* L. (oilseed rape) are the male sterility Lembke (MSL) in Europe and North America [5], Plant Genetic Systems (PGS) in North America [6], Ogura-Inra in North America and Europe [7], and Polimar cytoplasmic male sterility (Pol CMS) in China [8]. Compared with cytoplasmic male sterility (CMS), genic male sterility (GMS) has many advantages in heterosis utilization, including the facts that it represents stable and complete male sterility, it is easy to take advantage of the strong combination, it has a broad spectrum of restoration, there are no negative cytoplasmic effects and it includes diverse cytoplasmic sources [9]. GMS has a great potential in heterosis utilization, and some GMS systems have been used widely. However, recessive GMS (RGMS) has obvious disadvantages, i.e., it is difficult to obtain a whole sterile population, and 50% of fertile plants in the sterile line must be artificially removed during reproduction and seed production. The question of how to remove 50% of the fertile plants in the mother line with an economical and effective method is the main problem that has to be solved in the widespread application of nuclear sterility.

As an effective alternative to RGMS, dominant genic male sterility (DGMS) has been studied in *B. napus*. To date, some spontaneous DGMS mutants (Yi3A, 609A and TE5A) were systematically characterized. Genetic analyses confirmed that the sterility of Yi3A is controlled by one gene with three different alleles, which are *MS5^a^* (corresponding to the previous *Rf*)*, MS5^b^* (corresponding to the *Ms*) and *MS5^c^* (corresponding to the *ms*), respectively [10,11]. Individuals with the homozygous *MS5^b^* allele are male sterile, and the sterility is maintained by crossing with the lines carrying the homozygous *MS5^c^*. Another allele, *BnMS5^d^*, was derived from *BnMS5^a^* by mutagenesis and confers male sterility when it is homozygous [12]. Specifically, the *BnMS5^a^* allele is dominant over *BnMS5^b^* or *BnMS5^d^*, but *BnMS5^c^* is recessive to *BnMS5^b^* or *BnMS5^d^*. *BnMS5^a^BnMS5^a^* lines are referred to as restorer lines because they restore fertility when they crossed with *BnMS5^b^BnMS5^b^*/*BnMS5^d^BnMS5^d^* lines. *BnMS5^c^BnMS5^c^* lines are called temporary maintainer lines, because *BnMS5^b^BnMS5^c^*/*BnMS5^c^BnMS5^d^* plants are male sterile [11,12,13].

Our previous studies showed that 8029AB is a population with male fertile and sterile individuals, which can be segregated. It is a new type of DGMS sterile material whose fertility is controlled by a pair of multiple alleles (*BnMS5^a^/BnMS5^c^/BnMS5^e^*). The fertility was normal when *BnMS5^c^* was homozygous and *BnMS5^a^* was homozygous or heterozygous. Individuals with genotypes *BnMS5^a^* and *BnMS5^c^BnMS5^c^* were fertile, while plants with genotypes *BnMS5^e^BnMS5^e^* and *BnMS5^c^BnMS5^e^* were sterile. This material can simulate genic male sterility to achieve three lines of seed production: a two-type sterile line (AB line, genotype: *BnMS5^e^BnMS5^e^* and *BnMS5^a^BnMS5^e^*), a maintainer line (genotype: *BnMS5^c^BnMS5^c^*) and a restorer line (genotype: *BnMS5^a^BnMS5^a^*). The sterile line crosses with the maintainer line to produce a 100% sterile line; then, the 100% sterile line crosses with the restorer line to produce hybrids. Consequently, the problem that 50% fertile individuals must be removed from the parent line will be solved in three-line DGMS hybrid seed production.

The application of DGMS is still relatively limited because of the low efficiency of identifying complex genotypes with the desired and complicated agronomic traits, which is laborious and time consuming in the traditional breeding process. It is beneficial to develop molecular markers tightly linked to the sterility gene, maintainer gene and restorer gene for timely genotype identification in a DGMS three-line breeding system. Many AFLP markers linked to *BnMS5* have been identified; some of them were converted into SCAR markers [10,14,15]. Given the loose linkage relationship of some markers with *BnMS5*, the flanking SCAR markers BE10 and SCD8 restrict *BnMS5* to a 21 kb region containing six predicted functional genes [11]. Nevertheless, it remains unclear whether these markers are informative enough for molecular marker-assisted selection (MAS) in a DGMS three-line breeding system. Markers tightly linked with the sterility gene, maintainer gene and restorer gene, respectively, have not been developed. In this study, we report the genetics analysis of a new type of DGMS 8029A. We developed and validated two PARMS markers that were tightly linked to the sterility gene, maintainer gene and restorer gene, respectively. The application of these markers in high-throughput MAS of a new DGMS three-line hybrid system is demonstrated.

## 2. Materials and Methods

### 2.1. Plant Materials

**Figure 1:**
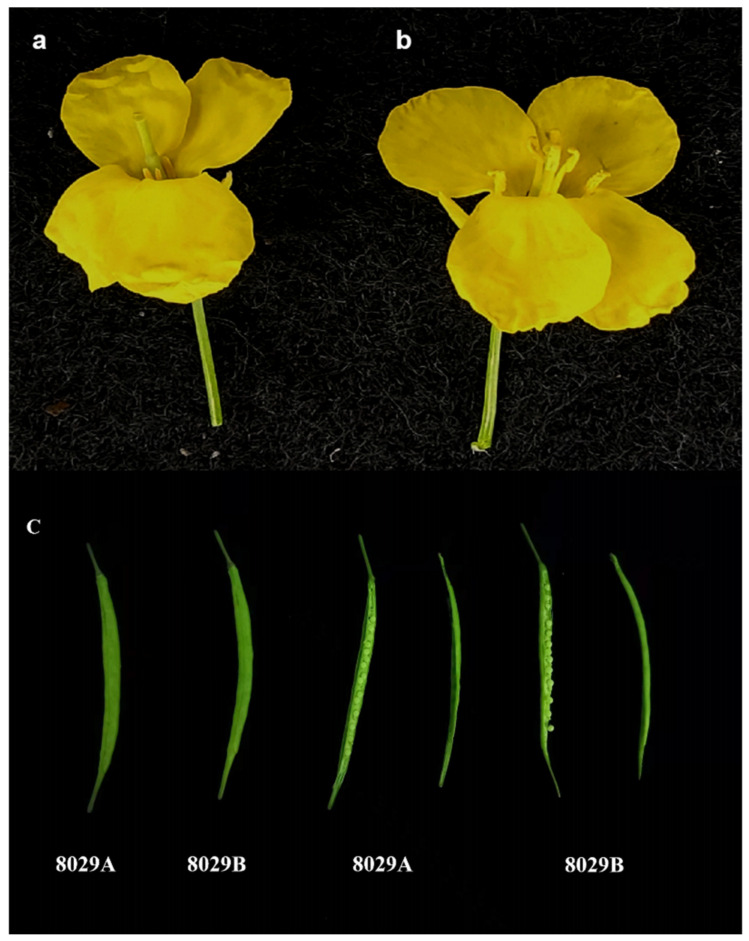
Flower morphology of 8029AB. (**a**) Male-sterile bud of 8029A. (**b**) Male-fertile bud of 8029B. (**c**) Siliques and seed setting of 8029A and 8029B.

8029AB is a newly bred, dominant genic male sterile (DGMS) two-type line in *Brassica napus* (Figure 1). The 8029A line (genotype: *BnMS5^e^BnMS5^e^*) was the sterile line, Zhongshuang11 (genotype: *BnMS5^c^BnMS5^c^*) was the maintainer line and 6449 (genotype: *BnMS5^a^BnMS5^a^*) was the restorer line. All the materials were procured from the Oil Crop Research Institute of the Chinese Academy of Agricultural Sciences. The seed setting of 8029A was normal under the conditions of artificially assisted pollination or natural pollination, indicating that the seed setting rate was not affected by the male sterile gene (Figure 1).

### 2.2. Genetic Analysis

One hybrid population (F_1_-1) was derived from the cross between the homozygous sterile plants of 8029A and the maintainer line Zhongshuang11. The BC_1_-1 line was obtained by backcrossing F_1_-1 to Zhongshuang11. The phenotype of F_1_-1 and the segregation ratio of the BC_1_-1 population were identified in the field to detect the genetic pattern of 8029A.

Furthermore, to analyze the genetic pattern of the restorer line 6449, F_1_-2, F_2_-2 and BC_1_-2 populations were constructed. F_1_-2 was obtained by crossing the homozygous sterile plants of 8029A with the restorer line 6449. The F_2_-2 population was derived from self-pollination of the F_1_-2 plants. BC_1_-2 was derived by crossing the homozygous sterile plants of 8029A with the F_1_-2 plants. The F_1_-2 phenotype and the segregation ratios of the F_2_-2 and BC_1_-2 populations were identified directly in the field.

### 2.3. DNA Extraction and PCR

The genomic DNA of young leaves, which were collected from individual plants at the seedling stage, was extracted by using the CTAB method, with some modifications. The DNA concentration was measured using a NanoDrop 2000 spectrophotometer (Thermo, Wilmington, Delaware, USA) and adjusted to 50 ng/μl.

The *BnMS5* gene of these *B. napus* lines was amplified via PCR. The master mix for the PARMS markers was purchased from Gentides Biotech Co., Ltd. (Wuhan, China). The 10 μl PCR reaction system contained 2× PARMS PCR reaction mix (containing two common fluorescent primers, PCR buffer, dNTP, Taq enzymes and internal standard ROX), 150 nM of each allele-specific primer, 400 nM of locus-specific primer, 50 ng of DNA template and 3.3 μl of ddH_2_O. The reaction conditions of PCR were optimized with parameters as follows: 95 ℃ for 5 min, followed by 10 cycles of 95 ℃ for 20 s and 65 ℃ (−0.8 ℃ per cycle) for 1 min, and 32 cycles of 95 ℃ for 20 s and 57 ℃ for 1 min and 72 ℃ for 5 min. The well plate was read using a TECAN infinite M1000 plate reader; SNP calling and plots were carried out using the online software snpdecoder (http://www.snpway.com/snpdecoder/ accesed on 17 June 2021) combining manual modification.

### 2.4. PARMS Markers Design and Markers Analysis

According to the sequence of *BnMS5 homologs*, *BnMS5^e^* was amplified in 8029A. *BnMS5^e^* was sequenced and compared with *BnMS5^a^* using BLAST. *BnMS5^e^* differs from *BnMS5^a^* by a few bases. Then, *BnMS5^e^* was transferred to the restorer line 6449. Part of transgenic plants were male sterile.

According to the sequence of *BnMS5^a^* (KX223882), *BnMS5^c^* (KX223881) and *BnMS5^e^* (Supplementary Materials Additional File S1), the SNP sites were excavated, and SNP markers were designed using Primer Premier 5.0. The PARMS molecular marker MS5-1F was designed based on the SNP variations from AC (*MS5^c^*) to GT (*MS5^a^*/*MS5^e^*). Additionally, MS5-2F was designed based on the SNP variations from T (*MS5^c^*/*MS5^e^*) to A (*MS5^a^*). The difference loci were placed in the position of the second base from the 3’ end of the primers; then, the fluorescent markers were added. MS5-1F and MS5-2F share a pair of reverse primers MS5-1R12. Two common primers which are consistent with the underlined parts of the two forward primers had different fluorescent markers at the tail. The underlined sequence was annealed to the oligonucleotides labeled with the FAM/HEX fluorophore (Table 1). The primers were commercially synthesized by Gentides Biotech Co., Ltd (Wuhan, China).

### 2.5. PARMS Markers Validate

F_1_-1 was obtained by crossing the sterile line 8029A and the maintainer line Zhongshuang11 and backcrossing the sterile plant with Zhongshuang11 as a recurrent parent, and finally, a (8029A×Zhongshuang11) a BC_4_-1 isolated population was obtained.

F_1_-2 was obtained via hybridization with the sterile line 8029A and restorer line 6449, and F_2_-2 was obtained by self-pollination of the F_1_-2 plants.

At the seedling stage, young leaves were taken from individual plants of BC_4_-1 (8029A×Zhongshuang 11) and the isolated population Pop (8029A×6449). To identify the genotype of individual plants in isolated populations, 100 individual plants were selected from the isolated populations BC_4_-1 (8029A×Zhongshuang 11) and Pop (8029A×6449), respectively, for PCR amplification using primers MS5-1Fc/MS5-1Ft/MS5-1R12 and MS5-2Ft/MS5-2Fa/MS5-1R12. At the flowering stage, the fertility of each individual plant in the isolated population BC_4_-1 (8029A×Zhongshuang 11) and Pop (8029A×6449) was investigated.

## 3. Results

### 3.1. Genetics Analysis of the Sterile Line 8029A and the Restorer Line 6449

The phenotype of the populations was thoroughly investigated in the field (Table 2). F_1_-1 individuals from the cross between 8029A and Zhongshuang11 were all male sterile, which were subsequently backcrossed with Zhongshuang11, resulting in a BC_1_-1 population that comprised 238 individuals (116 male sterile: 122 normal fertile). The segregation ratio of sterile individuals to fertile individuals showed the expected Mendelian inheritance ratio of 1:1 (χ^2^ = 0.15, *p* > 0.05). The data indicate that the sterility trait of 8029A is controlled by a single dominant nuclear allelic gene (*BnMS5 ^e^*).

F_1_-2 plants from the cross between the sterile line 8029A and the restorer line 6449 were all fertile, which were subsequently backcrossed with homozygous sterile plants of 8029A to obtain a BC_1_-2 population. The BC_1_-2 population was composed of 458 individuals (233 male sterile: 225 normal fertile). The segregation ratio of sterile individuals to fertile individuals showed the expected Mendelian segregation ratio of 1:1 (χ^2^ = 0.14, *p* > 0.05). The F_2_-2 population comprised 781 individuals, of which 208 plants were male sterile and 573 plants were normal fertile. The segregation ratio of sterile individuals to fertile individuals was consistent with the expected Mendelian segregation ratio of 1:3 (χ^2^ = 1.11, *p* > 0.05). The data indicate that the restorer line 6449 of 8029A is controlled by a pair of nuclear allelic genes.

### 3.2. Development of PARMS Markers

In total, six pairs of primer combinations were designed and identified. Finally, MS5-1Fc/MS5-1Ft/MS5-1R12 were identified as putative markers to distinguish *BnMS5^c^* and *BnMS5^a^*/*BnMS5^e^*. *BnMS5^a^* and *BnMS5^c^*/*BnMS5^e^* could be distinguished by MS5-2Ft/MS5-2Fa/MS5-1R12 (Table 1). All six genotypes, including *BnMS5aBnMS5a*, *BnMS5cBnMS5c*, *BnMS5eBnMS5e*, *BnMS5aBnMS5c*, *BnMS5aBnMS5e* and *BnMS5cBnMS5e*, could be distinguished by these two sets of primers (Table 3, Supplementary Materials Additional File S2).

### 3.3. Validation of the PARMS Markers

**Figure 2:**
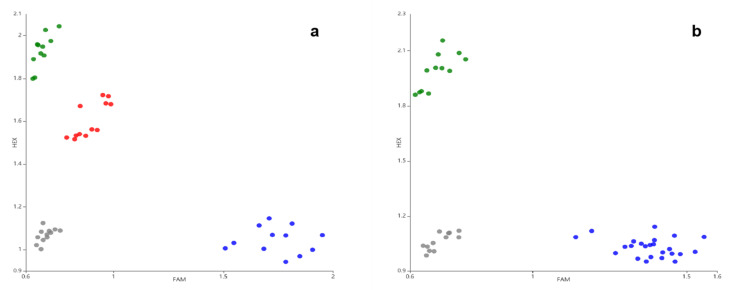
Genotyping of different rapeseed materials using the PARMS markers. (**a**) Fluorescence image amplified by MS5-1Fc/MS5-1Ft/MS5-1R12. The green, red and blue dots are the individuals with *BnMS5^a^BnMS5^a^*, *BnMS5^c^BnMS5^e^* and *BnMS5^c^BnMS5^c^* genotype, respectively. (**b**) Fluorescence image amplified by MS5-2Ft/MS5-2Fa/MS5-1R12. The green dots are the individuals with *BnMS5^a^BnMS5^a^* genotype. The blue dots are the individuals with *BnMS5^c^BnMS5^c^* and *BnMS5^c^BnMS5^e^* genotype. The gray dots are negative CK.

Thirty-six *B. napus* materials with genotypes *BnMS5^a^BnMS5^a^*, *BnMS5^c^BnMS5^c^* or *BnMS5^c^BnMS5^e^* were genotyped using the *BnMS5* functional molecular fluorescence markers MS5-1Fc/MS5-1Ft/MS5-1R12 and MS5-2Ft/MS5-2Fa/MS5-1R12, respectively (Figure 2). MS5-1Fc/MS5-1Ft/MS5-1R12 and MS5-2Ft/MS5-2Fa/MS5-1R12 matched well with the corresponding SNP to generate fluorescence signals and achieved the purpose of genotyping. The detected FAM fluorescence signal (blue dot) of MS5-1Fc/MS5-1Ft/MS5-1R12 and MS5-2Ft/MS5-2Fa/MS5-1R12 is a fertile plant with the *BnMS5^c^BnMS5^c^* genotype. The detected HEX fluorescence signal (green dot) of MS5-1Fc/MS5-1Ft/MS5-1R12 and MS5-2Ft/MS5-2Fa/MS5-1R12 is a fertile plant with the *BnMS5^a^BnMS5^a^* genotype. The FAM/HEX heterozygous state (red dot) of MS5-1Fc/MS5-1Ft/MS5-1R12 and FAM fluorescence signal (blue dot) of MS5-2Ft/MS5-2Fa/MS5-1R12 is a sterile plant with the *BnMS5^c^BnMS5^e^* genotype. The gray dot is negative CK (Figure 2).

To further validate these two pairs of PARMS markers in the detection of *BnMS5^a^, BnMS5^c^ and BnMS5^e^*, the BC_1_-1, F_2_ -2 and BC_1_-2 populations were selected to detect the polymorphism using MS5-1Fc/MS5-1Ft/MS5-1R12 and MS5-2Ft/MS5-2Fa/MS5-1R12. The amplification results of each plant were classified according to fluorescent signals. The genotypes of plants were classified based on a scatter diagram. The scatter diagram intuitively classified the corresponding genotype of each material. A comparison of the statistical data revealed that the phenotypic differentiation of the marker MS5-1Fc/MS5-1Ft/MS5-1R12 and MS5-2Ft/MS5-2Fa/MS5-1R12 was consistent with the field phenotypes statistics, indicating that the fluorescent molecular markers detected the SNP variations quickly and accurately.

## 4. Discussion

Several DGMS mutants were found in *B. napus*, and the inheritance of DGMS has been studied intensively [16,17,18,19,20]. However, due to their genetic complexity and low efficiency in the traditional breeding process, these DGMS materials have rarely been applied to large-scale commercial hybrid seed production. The 8029AB line is a new DGMS two-type line whose fertility is controlled by a pair of multiple alleles (*BnMS5^a^/BnMS5^c^/BnMS5^e^*). The sterility of 8029A was complete, and the seed setting rate was not affected by male sterile gene. Thus, this material can be used in a three-line approach for the seed production of rapeseed hybrids: a two-type sterile line (AB line, genotype: *BnMS5^e^BnMS5^e^* and *BnMS5^a^BnMS5^e^*), a maintainer line (genotype: *BnMS5^c^BnMS5^c^*) and a restorer line (genotype: *BnMS5^a^BnMS5^a^*). The sterile line crosses with the maintainer line to produce 100% sterile population, and then, the 100% sterile line and restorer line are used to produce hybrids.

The development of new DGMS lines as well as maintainer lines and excellent restorer lines through conventional backcrossing and selfing is time consuming and labor intensive. To facilitate the three-line breeding process, many attempts have been made to develop a molecular marker linked to *BnMS5*. Two AFLP markers were firstly identified at either side of the *BnMS5^b^* allele, at a distance of 3.7 and 5.9 cM, respectively [10]. Subsequently, several SCAR markers linked to *BnMS5^a^* were developed, which followed a dominant inheritance mode, and it is impossible to distinguish between heterozygous and homozygous individuals at the *BnMS5^a^* site [14]. Meanwhile, using DGMS line 609AB, the marker linked to *BnMS5^a^* was conversed into a marker linked to *BnMS5^b^*, providing proof of allelism between previously *Ms* and *Mf* genes [21]. Later, two closer markers with respective distances of 0.1 and 1.2 cM to the *BnMS5* locus were reported [22]. Given the loose linkage relationship of some markers with *BnMS5*, the flanking SCAR markers BE10 and SCD8 restrict *BnMS5* to a 21 kb region containing six predicted functional genes [11]. Nevertheless, it remains unclear whether these markers are informative enough for MAS in a DGMS three-line breeding system. What is more, most molecular markers are random DNA markers which might be lost during recombination [23].

Gel-electrophoresis-based markers such as SSR (simple sequence repeat), AFLP (amplified fragment length polymorphism), SCAR (sequence-characterized amplified region) and CAPS (cleaved amplified polymorphic sequence) have been used in molecular breeding [24]. However, electrophoresis is time consuming, laborious and has a low throughput. To break this bottleneck, the gel-free and automated SNP marker system with high throughput, such as sequencing, was extensively developed. SNP markers have been developed and employed in rapeseed, maize, rice and bean [25,26,27]. Nevertheless, it is too expensive for most breeders to afford [18,28]. In contrast, PARMS (Gentides, China) detects SNPs based on fluorescence detection. It is an easy, robust and low-cost genotyping system which is adaptable to rapeseed, rice, maize and wheat in mapping and molecular breeding [29,30,31]. In our study, we successfully developed the PARMS molecular markers of *BnMS5,* which are highly efficient in genotyping for *BnMS5* (Figure 2). Hence, they will greatly accelerate DGMS three-line breeding of *BnMS5* in *B. napus*.

## 5. Conclusions

In this study, we identified PARMS markers, which were designed according to the sequence of *BnMS5^a^*, *BnMS5^c^* and *BnMS5^e^*. The PARMS markers were tightly linked to *BnMS5^a^, BnMS5^c^* and *BnMS5^e^,* respectively, which can predict the presence or absence of *BnMS5^a^/BnMS5^c^/BnMS5^e^* effectively. Consequently, marker-assisted selection can be carried out in the early generation to shorten the breeding period and improve the breeding efficiency, so as to realize the large-scale commercial production of DGMS three-line hybrid seed in rapeseed.

## Figures and Tables

**Table 1:** The PARMS markers used in the detection of *BnMS5^a^, BnMS5^c^* and *BnMS5^e^*.

Marker Name	Sequences	Remarks
*Ms5-1Fc*	GAAGGTGACCAAGTTCATGCTCCAGCTACCTCCTCCTTTGTTAC	Forward primer
*MS5-1Ft*	GAAGGTCGGAGTCAACGGATTCAGCTACCTCCTCCTTTGTTGT	Forward primer
*MS5-2Ft*	GAAGGTGACCAAGTTCATGCTCTTGTTATATCTCAAGACCTAAAGGTTT	Forward primer
*MS5-2Fa*	GAAGGTCGGAGTCAACGGATTCTTGTTATATCTCAAGACCTAAAGGTTA	Forward primer
*MS5-1R12*	AATTAATTACAAAGAAAAGCGCG	Reverse primers
#1	GAAGGTGACCAAGTTCATGCT-FAM	Common primer labeled with the FAM fluorophore
#2	GAAGGTCGGAGTCAACGGATT-HEX	Common primer labeled with the HEX fluorophore

**Table 2:** Genetic analysis of inheritance sterility/fertility characters in rapeseed lines.

Population	Total Individuals	Sterile Individuals	Fertile Individuals	Sterile: Fertile	Expected Mendelian Segregation Ratio	χ^2^
BC1-1	238	116	122	0.95:1	1:1	0.15, *p* > 0.05
F2-2	781	208	573	1:2.8	1:3	1.11, *p* > 0.05
BC1-2	458	233	225	1.0:1	1:1	0.14, *p* > 0.05

**Table 3:** PARMS primer sets for genotyping.

	*MS5^a^MS5^a^*	*MS5^c^MS5^c^*	*MS5^e^MS5^e^*	*MS5^a^MS5^c^*	*MS5^a^MS5^e^*	*MS5^c^MS5^e^*
MS5-1F	HEX (Green)	FAM (Blue)	HEX (Green)	HEX/FAM (Red)	HEX (Green)	HEX/FAM (Red)
MS5-2F	HEX (Green)	FAM (Blue)	FAM (Blue)	HEX/FAM (Red)	HEX/FAM (Red)	FAM (Blue)

## Data Availability

Not applicable.

## Supplementary Materials

The following are available online at https://www.mdpi.com/article/10.3390/plants11030421/s1, Additional File S1: cDNA of *BnMS5e*, Additional File S2: Classification data of PARMS primer sets for genotyping according to the fluorescent signals.
